# A Spatial and Temporal Gradient of Fgf Differentially Regulates Distinct Stages of Neural Development in the Zebrafish Inner Ear

**DOI:** 10.1371/journal.pgen.1003068

**Published:** 2012-11-15

**Authors:** Shruti Vemaraju, Husniye Kantarci, Mahesh S. Padanad, Bruce B. Riley

**Affiliations:** Biology Department, Texas A&M University, College Station, Texas, United States of America; Harvard Medical School, United States of America

## Abstract

Neuroblasts of the statoacoustic ganglion (SAG) initially form in the floor of the otic vesicle during a relatively brief developmental window. They soon delaminate and undergo a protracted phase of proliferation and migration (transit-amplification). Neuroblasts eventually differentiate and extend processes bi-directionally to synapse with hair cells in the inner ear and various targets in the hindbrain. Our studies in zebrafish have shown that Fgf signaling controls multiple phases of this complex developmental process. Moderate levels of Fgf in a gradient emanating from the nascent utricular macula specify SAG neuroblasts in laterally adjacent otic epithelium. At a later stage, differentiating SAG neurons express Fgf5, which serves two functions: First, as SAG neurons accumulate, increasing levels of Fgf exceed an upper threshold that terminates the initial phase of neuroblast specification. Second, elevated Fgf delays differentiation of transit-amplifying cells, balancing the rate of progenitor renewal with neuronal differentiation. Laser-ablation of mature SAG neurons abolishes feedback-inhibition and causes precocious neuronal differentiation. Similar effects are obtained by Fgf5-knockdown or global impairment of Fgf signaling, whereas Fgf misexpression has the opposite effect. Thus Fgf signaling renders SAG development self-regulating, ensuring steady production of an appropriate number of neurons as the larva grows.

## Author Summary

Neurons of the statoacoustic ganglion (SAG), which innervate the inner ear, are derived from neuroblasts originating from the floor of the otic vesicle. Neuroblasts quickly delaminate from the otic vesicle to form dividing progenitors, which eventually differentiate into mature neurons of the SAG. Fgf has been implicated in initial neuroblast specification in multiple vertebrate species. However, the role of Fgf at later stages remains uncertain, because previous studies have not been able to evaluate the effects of changing levels of Fgf, nor have they been able to clearly distinguish the effects of Fgf at different stages of SAG development. We have combined conditional loss of function, misexpression, and laser-ablation studies in zebrafish to elucidate how graded Fgf coordinates distinct steps in SAG development. Initially moderate Fgf in a spatial gradient specifies neuroblasts within the otic vesicle. Later, mature SAG neurons express Fgf5 and, as additional neurons accumulate outside the otic vesicle, rising levels of Fgf terminate further specification. Elevated Fgf also slows maturation of progenitors, maintaining a stable progenitor pool in which growth and differentiation are evenly balanced. This feedback facilitates steady production of new neurons as the animal grows through larval and adults stages.

## Introduction

Neurons of the VIII^th^ cranial ganglion, or the statoacoustic ganglion (SAG), innervate sensory hair cells in the inner ear. These bipolar neurons relay auditory and vestibular information to the hindbrain. During development, SAG precursors (neuroblasts) originate in the floor of the otic vesicle during a relatively brief window of time. Newly specified neuroblasts soon delaminate from the floor of the otic vesicle before continuing development outside the ear. Neuroblast specification requires the bHLH transcription factor *neurogenin1* (*neurog1*) [1], [2]. Expression of *neurog1* is transient and is followed by strong upregulation of *neurod*, which encodes a related bHLH transcription factor required for completing neuronal differentiation [1], [3]. After delamination, neuroblasts migrate a short distance to become situated between the hindbrain and otic vesicle and undergo a transient phase of proliferation to expand the precursor population [4]–[7]. This phase, termed transit-amplification, is characterized by co-expression of *neurod* and proliferation markers [8]. Neuroblasts eventually exit the cell cycle and differentiate into mature neurons.

Numerous studies suggest a role for Fgf in otic neurogenesis. In chick, Fgf10 is expressed in the neurosensory domain of the otic placode and promotes neuroblast specification [4]. Elevating Fgf2 or Fgf8 increases the number of SAG neurons [9], [10], though the mechanism of action in these cases has not been determined. In mouse, *Fgf3* is also expressed in the neurosensory domain, and SAG development is impaired in *Fgf3* null mutants [11]. In zebrafish, *fgf3* and *fgf8* are prominently expressed in the developing utricular macula adjacent to the neurogenic domain [12], [13], and impairment of *fgf8* causes a reduction in SAG markers [12], [14]. Also in zebrafish, mutations that expand the domain of *fgf3* expression in the hindbrain cause a corresponding expansion of anterior markers in the otic vesicle, including markers of the utricular macula and neurogenic domain [15], [16]. Unfortunately, interpretation of these mutant phenotypes in mouse and zebrafish is clouded because morphogenesis of the inner ear is significantly altered. Additionally, previous studies have not been able to clearly distinguish effects of changing Fgf levels on different stages of SAG development.

Here we study the development of SAG and its regulation by Fgf by conditionally manipulating Fgf signaling levels. We show that Fgf signaling differentially controls distinct stages of otic neurogenesis. A moderate level of Fgf is necessary for the initial specification of neuroblasts in the floor of the otic vesicle, whereas high levels of Fgf inhibit specification. During later stages of SAG development, Fgf5 expressed by mature SAG neurons serves two roles. First, upon accumulation of sufficient mature neurons the phase of specification is terminated. Second, ongoing Fgf signaling delays the differentiation of SAG precursor cells. This ensures maintenance of progenitors and steady production of an appropriate number of mature neurons.

## Results

### Development of the statoacoustic ganglion (SAG)

**Figure 1:**
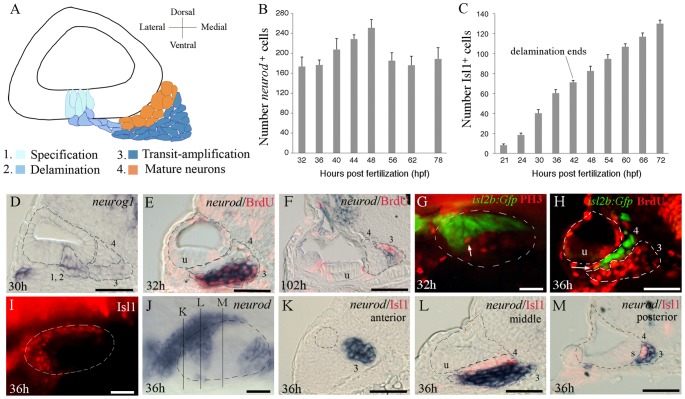
Development of Statoacoustic Ganglion (SAG). (A) Illustration showing the various stages of SAG development. Neuronal precursors (neuroblasts) are specified (1) and delaminate from (2) the floor of the otic vesicle. Neuroblasts undergo a phase of transit-amplification (3) wherein they migrate to a position between the otic vesicle and hindbrain as they continue to proliferate. Neuroblasts finally differentiate into mature neurons (4). (B) Total number of delaminated *neurod^+^* cells within the SAG counted from serial sections at the indicated times (mean ± standard deviation, n = 2 or greater for each time point). (C) total number of Islet-1-positive SAG neurons at the indicated times (mean of total number ± standard deviation, n = 20 for each time point). (D) *neurog1* expression at 30 hpf. (E, F) Co-staining for *neurod* (blue) and BrdU (red) in embryos exposed to BrdU for 6 hours starting at 26 hpf (E) and 96 hpf (F), and then fixed at 32 hpf and 102 hpf, respectively. (G) Co-staining for *isl2b:Gfp* (green) and phospho-histone H3 (PH3, red) at 32 hpf. Only one mitotic cell (arrow) is seen in the vicinity of the SAG. (H) Co-staining for Islet1 (green) and BrdU (red) at 36 hpf. Only one double-stained cell is visible (arrow). (I–M) Expression of *neurod* (blue) and Islet-1 (red) at 36 hpf. Mature neurons are labeled with Islet-1 (I) and delaminated progenitor cells express *neurod* (J). Positions of section-planes in K–M are indicated in (J). (K–L) Transverse sections passing through the anterior (K), middle (L) and posterior (M) regions of the SAG show mostly complementary patterns of *neurod* and Islet-1. The outer edge of the otic vesicle is outlined in all panels. SAG cells in stages 1–4 of development are indicated in sections, and the position of the utricular macula (u) is indicated. Images of whole-mount specimens (G, I, J) show dorsolateral (G, I) and dorsal (J) views with anterior to the left. Images of transverse sections (C–F, H, K–M) show dorsal to the top and lateral to the left. Scale bar, 25 µm.

The paradigm for otic neurogenesis, as formalized in several recent reviews [17], [18], involves a sequential process of specification, delamination, proliferative expansion and differentiation of precursor cells to form the mature SAG. The general features of this process appear to be conserved in zebrafish, shown schematically in Figure 1A. In zebrafish, SAG neuroblasts are initially specified in the floor of the late placode/nascent vesicle as early as 16 hpf (14 somites) and express *neurog1* [1], [19]. Neuroblasts begin to delaminate and accumulate outside the otic vesicle by 17 hpf. The reiterative process of specification and delamination peaks at 24 hpf, continues at a more moderate pace through 30 hpf (Figure 1D), then declines sharply and stops entirely by 42 hpf [20]. Expression of *neurog1* is only transient. As neuroblasts delaminate they lose expression of *neurog1* and initiate expression of *neurod* [1], [21]. At this point SAG precursors enter a phase of transit-amplification, as shown by co-labeling with *neurod* expression and BrdU incorporation (Figure 1E, 1F). *neurod* ^+^ cells continue to proliferate through at least 4 days post fertilization (dpf), the latest stage examined (Figure 1F). Surprisingly, staining with anti-phospho histone H3 shows that there are typically only 1–2 mitotic cells in the SAG at any time between 24 and 50 hpf (Figure 1G, and data not shown), indicating that transit-amplifying cells cycle relatively slowly. Summing *neurod* ^+^ cells in serial sections revealed that the number of transit-amplifying cells remains relatively constant after 30 hpf, with a transient peak at 48 hpf followed by a return to steady state of 180–200 cells through 78 hpf (Figure 1B). As precursor cells begin to differentiate they exit the cell cycle and lose expression of *neurod* (Figure 1E, 1F) and initiate expression of *isl1/2* genes (Figure 1H, 1I) [3]. The first mature Isl1^+^ neurons appear by 20 hpf and almost immediately begin to extend processes to peripheral and central targets (data not shown). Co-staining for BrdU and Gfp in *isl2b:Gfp* transgenic embryos [22] confirms that relatively few mature neurons incorporate BrdU (Figure 1H). The number of Isl1^+^ neurons increases linearly at a rate of 2–2.5 neurons per hour through at least 72 hpf, despite the cessation of specification and delamination at 42 hpf (Figure 1C). The steady increase in mature neurons after 42 hpf presumably reflects ongoing differentiation from the slowly cycling pool of transit-amplifying cells. The slow mitotic rate amongst precursors presumably counterbalances production of new neurons, thereby maintaining a relatively stable transit-amplifying pool.

To clarify the spatial relationship between transit-amplifying and mature SAG cells, we examined sections co-stained for *neurod* and Isl1 (Figure 1I–1M). The most mature neurons accumulate in immediate contact with the ventromedial surface of the ear, while *neurod* ^+^ cells undergoing transit-amplification reside more distally (Figure 1L, 1M). By 36 hpf the SAG also develops a more complex spatial distribution, forming three distinct regions along the anterior-posterior axis: The anterior-most region abuts the front end of the otic vesicle and contains only *neurod* ^+^ precursors (Figure 1K), although mature neurons accumulate in this region at later stages (see below). The middle region forms a broad mass spreading mediolaterally beneath the utricular region and contains complementary domains of *neurod* ^+^ cells and Isl1^+^ cells (Figure 1L). The posterior-most region forms as a narrow finger of Islet^+^ cells and abutting *neurod* ^+^ cells extending along the medial surface of the otic vesicle to the level of the saccular macula (Figure 1M). Segregation of neurons into these three AP domains reflects emergence of the topological pattern of innervation of the inner ear: Specifically, Sapède and Pujades [23] reported that anteroventral SAG neurons (corresponding to the anterior and middle regions reported here) predominantly innervate the utricular macula and to a lesser degree anterior and lateral cristae, whereas posterior-medial SAG neurons (corresponding to the posterior region reported here) predominantly innervate the saccular macula and to a lesser degree the posterior crista.

### A general model of SAG regulation and manipulation of Fgf signaling

**Figure 2:**
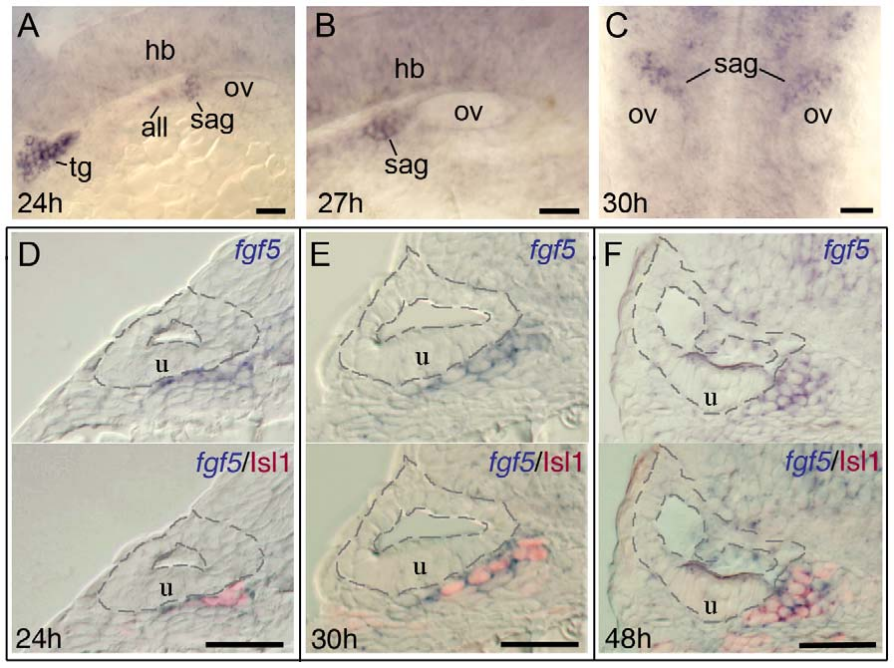
Mature SAG neurons express *fgf5*. (A–C) Wholemount embryos showing lateral views of *fgf5* expression at 24 hpf (A) and 27 hpf (B) and a dorsal view at 30 hpf (C). During these stages, *fgf5* expression marks the trigeminal ganglion (tg), anterior lateral line ganglion (all) and SAG, and there is also weak diffuse expression in the developing hindbrain (hb). There is no detectable staining in the otic vesicle (ov). (D–F) Transverse sections (dorsal to the top and lateral to the left) of specimens co-stained for *fgf5* (blue) and Islet-1 (red) at 24 hpf (D), 30 hpf (E) and 48 hpf (F). Sections pass through the middle portion of the SAG at the level of the utricular macula (u). The inner and outer surfaces of the otic vesicle are outlined. Co-labeling confirms that *fgf5* expression in the SAG is restricted to mature neurons. Scale bar, 25 µm. During mid-somitogenesis stages *fgf5* is diffusely expressed throughout the neural tube and strongly marks the developing trigeminal ganglion (not shown).

**Figure 3:**
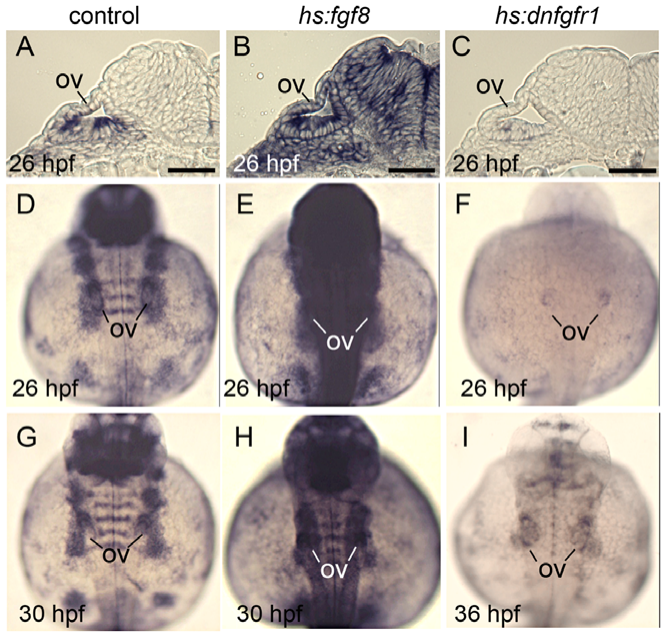
Effects of transgene activation on expression of the Fgf reporter *etv5b*. All embryos were heat shocked for 30 minutes beginning at 24 hpf. Wild-type and *hs:fgf8* embryos were heat shocked at 39°C and *hs:dnfgfr1* embryos were heat shocked at 38°C. (A–C) Cross sections showing *etv5b* expression in wild-type (A), *hs:fgf* (B) and *hs:dnfgfr1* (C) embryos at 26 hpf. (D–I) Dorsal views (anterior up) of wholemounts showing *etv5b* expression in wild-type (D, G), *hs:fgf8* (E, H) and *hs:dnfgfr1* (F, I) embryos at the indicated times. The otic vesicle (ov) is marked. Expression of *etv5b* remains elevated in *hs:fgf8* embryos for at least 6 hours after heat shock, whereas *etv5b* expression is downregulated in *hs:dnfgfr1* for at least 12 hours after heat shock. Scale bar, 25 µm.

The data presented in subsequent sections support a model in which changing levels of Fgf differentially affect SAG development: Initially, moderate Fgf from nearby cells promotes neuroblast specification in the otic vesicle. Subsequently, Fgf levels rise in part because mature SAG neurons specifically express Fgf5 and accumulate just outside the otic vesicle (Figure 2). Elevated Fgf then terminates further specification/delamination and also inhibits maturation of transit-amplifying precursors. Manipulation of Fgf to test this model was achieved by specifically knocking down *fgf5* (described below) and more generally by using two heat shock inducible transgenic lines, *hs:fgf8* and *hs:dnfgfr1* (dominant-negative Fgf receptor), to increase or decrease Fgf signaling, respectively [24], [25]. To document the efficacy of these transgenic lines, we examined expression of the Fgf-feedback gene *etv5b* (previously *erm*) [26], [27] following activation of the transgenes. Strong activation of *hs:fgf8* by heat shocking embryos at 24 hpf (30 minutes at 39°C) led to a detectable increase in *etv5b* levels by the end of the heat shock period (not shown), with maximal *etv5b* seen throughout the embryo by 26 hpf (Figure 3B, 3E). *etv5b* levels remained elevated through 30 hpf (Figure 3H) and subsequently returned to normal. In contrast, strong activation of *hs:dnfgfr1* at 24 hpf (30 minutes at 38°C) led to marked reduction of *etv5b* expression throughout the embryo by 25 hpf (not shown), and complete loss by 26 hpf (Figure 3C, 3F). Expression first began to return by 36 hpf, though levels were still well below normal at that time (Figure 3I). These transgenes were subsequently used to assess the effects of changing Fgf signaling levels at different stages of SAG development.

### Fgf regulates SAG specification in a dose-dependent manner

**Figure 4:**
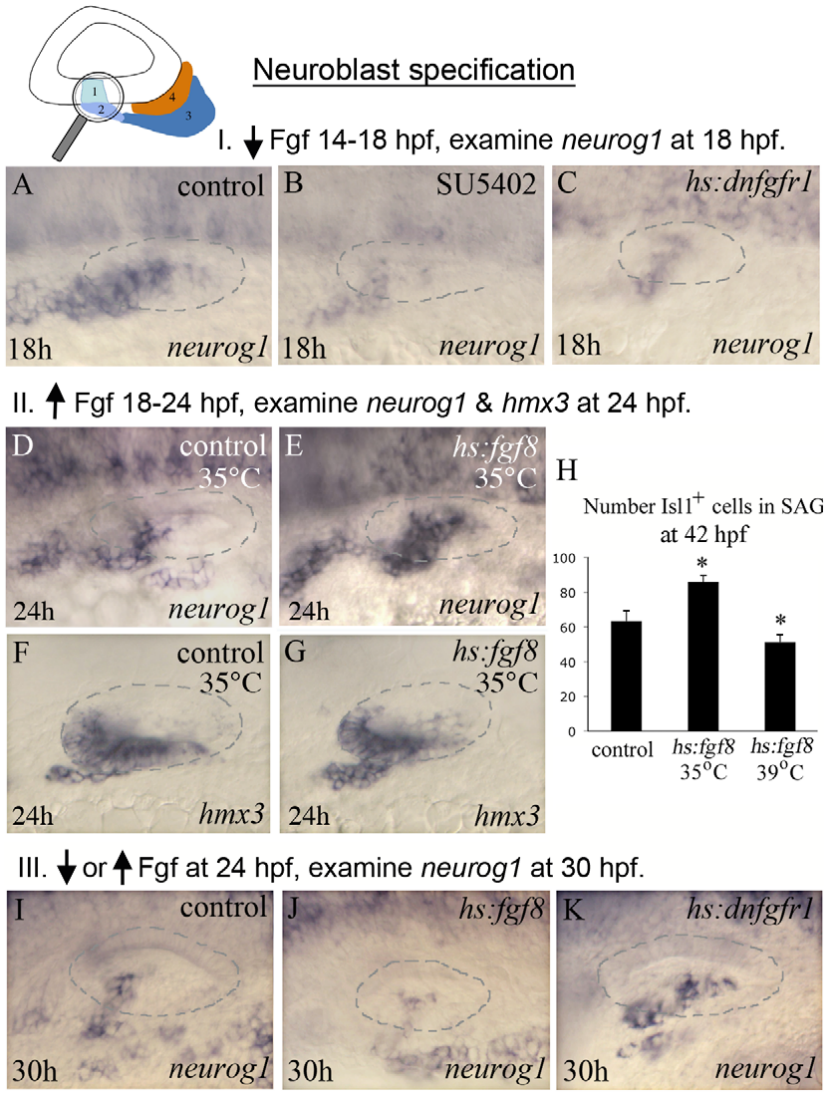
Fgf regulates neuroblast specification. The icon at the top of the figure indicates that analysis focuses on initial stages of neuroblast formation, as normally marked by *neurog1* expression. Experimental manipulations in groups I, II and III are briefly summarized at the tops of the corresponding data panels. (A–C) Experiment I, *neurog1* expression at 18 hpf in a control (A), SU5402 inhibitor treated (B) and *hs:dnfgfr1/+* transgenic embryo heat shocked for 30 minutes at 38°C beginning at 14 hpf. Blocking Fgf strongly reduces expression of *neurog1*. (D–G) Experiment II, expression of *neurog1* (D–E) and *hmx3* (F–G) in control and *hs:fgf8/+* embryos heat shocked at 35°C for 6 hours, from 18 hpf until 24 hpf. This regimen results in weak overexpression of Fgf8, which at this stage enhances expression of *neurog1*. (H) Experiment II, total number of Islet1-positive cells in the SAG (mean and standard deviation, n = 15) at 42 hpf following heat shock activation of *hs:fgf8* at 18 hpf at indicated temperatures. Weak misexpression of Fgf8 (35°C) increases production of SAG neurons whereas strong misexpression of Fgf8 (39°C) reduces production of SAG neurons. *p<0.001 in comparison to the control, analyzed with Student's *t* test. (I–K) Group III, *neurog1* expression at 30 hpf following heat shock at 24 hpf in control embryos (I), *hs:fgf8/+* embryos heat shocked at 39°C for 30 minutes to strongly over-express Fgf (J) and *hs:dnfgfr1/+* embryos heat shocked for 2 hours at 35°C and then shifted to 33°C to maintain low level inhibition of Fgf signaling (K). At this stage, weak impairment of Fgf enhances *neurog1* expression, consistent with the idea that Fgf levels normally increase during development and become inhibitory for neuroblast specification. All images show dorsolateral views with anterior to the left, and the otic vesicle is outlined.

Several Fgfs expressed in tissues near the developing SAG have been implicated in establishing a neurogenic domain in the ear [28]. In zebrafish, *fgf3* is expressed in the adjacent hindbrain through placodal stages and later helps initiate expression of *fgf3* and *fgf8* in the nascent utricular macula by 18 hpf [15]. We have hypothesized that sensory-neural patterning is spatially coordinated by a lateral gradient of Fgf, with high levels initiating sensory development in the medial half of the otic placode – e.g. closest to the Fgf source [13], and lower levels specifying the neurogenic domain in laterally adjacent otic epithelium. We previously documented a stringent requirement for Fgf in sensory development [13] and here we focused on the requirement for Fgf in neurogenic specification. To bypass the early requirements of Fgf during otic induction we used the chemical inhibitor, SU5402, to block Fgf signaling at later stages of otic development. Embryos treated with 100 µM SU5402 from 14 hpf −18 hpf showed a strong reduction in *neurog1* expression (Figure 4A, 4B). Likewise, impairment of Fgf signaling by strongly activating *hs:dnfgfr1* [25] (38°C for 30 minutes) showed similar results (Figure 4C). Blocking Fgf from this early stage caused widespread cell death at later stages, precluding analysis of SAG maturation. Nevertheless, these data confirm that normal specification of the neurogenic domain requires Fgf signaling. To test the hypothesis that SAG neuroblasts are specified by a specific lower level of Fgf in a signaling gradient, we manipulated Fgf levels using *hs:fgf8* [25]. The level of *hs:fgf8* activity can be adjusted by heat shocking at different temperatures [29]. To provide a broad shelf of low Fgf signaling, embryos were incubated at 35°C from 18 hpf to 24 hpf. This caused a marked upregulation and expansion of *neurog1* expression (Figure 4D, 4E). Additionally, there was a notable increase in the number of delaminating neuroblasts as seen by *hmx3* expressing cells leaving the vesicle (Figure 4F, 4G). By 42 hpf, the number of Isl1^+^ cells in the mature SAG had increased by 37% over the control (Figure 4H, 63±6.0 Isl1^+^ cells in control embryos compared to 86±3.6 in *hs:fgf8* transgenic embryos, n = 15). To evaluate the effects of a higher level of Fgf, *hs:fgf8* embryos were maximally induced by heat shocking them at 39°C for 30 minutes beginning at 18 hpf. Under these conditions, *neurog1* expression was reduced for several hours following heat shock but recovered to near normal by 24 hpf (data not shown). However, the number of mature Isl1^+^ neurons at 42 hpf was reduced by 20% (51±4.2 Isl1^+^ cells, n = 15; Figure 4H). As summarized in Table 1, these data support the idea that Fgf acts in a concentration-specific manner, with lower levels promoting neuroblast specification and higher levels inhibiting specification.

**Table 1:** Effects of altering Fgf on neuroblast specification.

Condition and stage	*neurog1* domain	Figure
*hs:fgf8*-high (39°C), 18 hpf	reduced 20 hpf	-
*hs:fgf8*-low (35°C), 18 hpf	enlarged 24 hpf	4E
*hs:dnfgfr1*-high (38°C), 14 hpf	reduced 18 hpf	4C
SU5402 (100 µM), 14 hpf	reduced 18 hpf	4B
*hs:fgf8*-high (39°C), 24 hpf	reduced 30 hpf	4J
*hs:fgf8*-low (35°C), 24 hpf	reduced 30 hpf	-
*hs:dnfgfr1*-high, 24 hpf	reduced 30 hpf	4K
*hs:dnfgfr1*-low (35°C), 24 hpf	enlarged 30 hpf	-
*fgf5*-MO, 1-cell	enlarged 30,36 hpf	6E–6G
*fgf5*-MO+*hs:fgf8* (39°C), 24 hpf	normal 36 hpf	6H
Ablate mature SAG 22, 25 hpf	enlarged 30 hpf	6L

Because the rate of neuroblast specification and delamination peaks at 24 hpf, we examined the effects of Fgf misexpression during this stage. As before, maximal activation of *hs:fgf8* (39°C) at 24 hpf reduced expression of *neurog1* in the ear by 30 hpf (Figure 4I, 4J). However, in contrast to earlier stages, low level activation of *hs:fgf8* (35°C) at 24 hpf reduced *neurog1* expression by 30 hpf (data not shown). Fully blocking Fgf by strong activation of *hs:dnfgfr1* (38°C) at 24 hpf also diminished *neurog1* expression by 30 hpf (data not shown), in keeping with the requirement for Fgf in neuroblast specification. However, weak attenuation of Fgf signaling by activating *hs:dnfgfr1* at a low level (35°C for 2 hours followed by incubation at 33°C) expanded the *neurog1* expression domain at 30 hpf (Figure 4K). Overall these data (summarized in Table 1) support the hypothesis that a specific low-to-moderate level of Fgf promotes neuroblast specification at both early and later stages, and either a high level of Fgf signaling or complete blockage of Fgf signaling impairs this process. At later stages, however, the process of specification becomes increasingly sensitive to inhibition by elevated Fgf. This likely reflects the finding that the level of Fgf increases during development, as described in the next section.

### *fgf5* from mature neurons inhibits neuroblast specification

**Figure 5:**
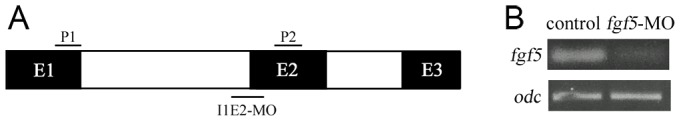
Efficacy of *fgf5* splice-blocking MO. (A) Schematic of *fgf5* mRNA showing intron-exon structure (not to scale). Binding sites for splice-blocking morpholino at intron1-exon2 junction (I1E2-MO) and PCR primers for RT-PCR (forward P1, reverse P2) are shown. (B) RT-PCR results showing the efficacy of I1E2-MO. *fgf5* transcript levels are severely reduced in *fgf5* morphants at 24 hpf. *odc* transcript level was used as a constitutive control.

Because SAG specification becomes increasingly sensitive to inhibition by elevated Fgf, we hypothesized that the process of neuroblast specification is normally terminated by a developmental increase in local Fgf signaling. To explore this possibility, we surveyed expression of all known *fgf* genes in zebrafish and identified *fgf5* as a strong candidate for a feedback regulator of SAG development. During mid-somitogenesis stages *fgf5* is diffusely expressed throughout the neural tube and strongly marks the developing trigeminal ganglion (not shown). As mentioned above, *fgf5* shows relatively specific expression in mature SAG neurons, and several other cranial ganglia, by 24 hpf and this pattern is maintained through at least 48 hpf (Figure 2). No expression is detected in the otic vesicle or other nearby tissues. We tested the role of Fgf5 by injecting morpholino oligomers to block translation (*fgf5tb-*MO) or to disrupt splicing at the intron1-exon2 splice junction (*fgf5i1e2*-MO). Injection of either MO yielded identical phenotypes: Morphants showed highly specific and fully penetrant enhancement of SAG specification and maturation, as described below, but otherwise there were no other detectable changes in embryo morphology nor was there a detectable increase in cell death. For most experiments reported here, we show results obtained with *fgf5i1e2*-MO, which proved to be highly effective in reducing mature *fgf5* transcript levels (Figure 5A, 5B).

**Figure 6:**
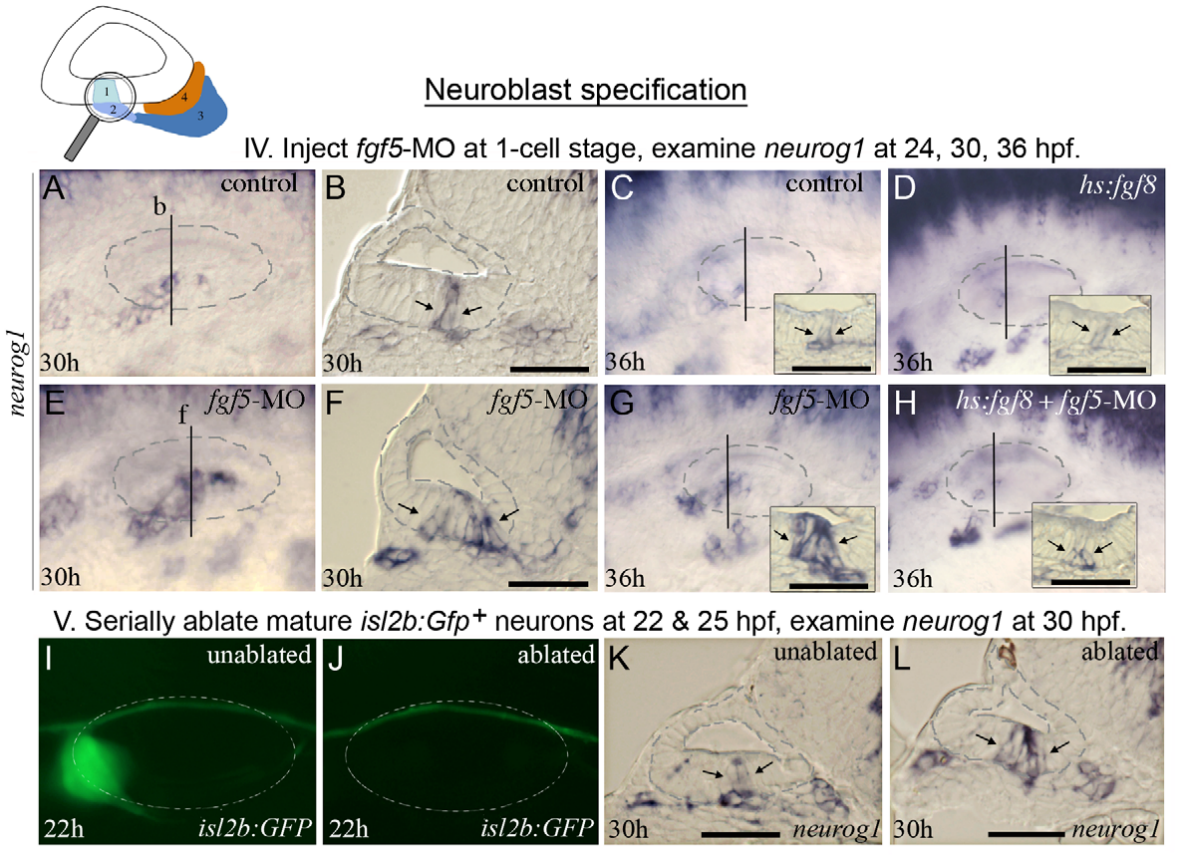
*fgf5* from mature neurons terminates the phase of neuroblast specification. The icon at the top of the figure indicates that analysis focuses on initial formation of neuroblasts. Experimental manipulations in groups IV and V are briefly summarized at the tops of the corresponding data panels. (A–H) Expression of *neurog1* in control embryos (A–C), a *hs:fgf8* embryo (D), *fgf5* morphants (E–G), and a *hs:*fgf8 embryo injected with *fgf5*-MO (H) at the indicated stages. Transgenic embryos (D, H) were heat shocked for 30 minutes at 39°C beginning at 24 hpf. Vertical lines in (A, C–E, G, H) indicate the plane of transverse sections in (B, F, and insets in C, D, G and H). (I–L) Expression of *isl2b:Gfp* at 22 hpf (I, J) and *neurog1* at 30 hpf (K, L) in a specimen in which mature (*fgf5*-expressing) neurons were laser-ablated. The same specimen is shown in all panels. Mature SAG neurons expressing *isl2b:Gfp* were serially ablated on the left side at 22 hpf (J) and 25 hpf (not shown), and the embryo was fixed and sectioned at 30 hpf to examine *neurog1* expression (L). Images of the unablated right side (I, K) were inverted to facilitate comparison. The surface of the otic vesicle is outlined in all panels. Arrows in sections indicate the edges of *neurog1* domain in the otic floor. Note that the amount and duration of delamination of *neurog1^+^* neuroblasts is strongly enhanced by knockdown of *fgf5* (F, G) or ablation of mature neurons (L). Activation of *hs:fgf8* reverses the effects of *fgf5-*MO (H). Scale bar, 25 µm. Transverse sections are shown with lateral to the left and dorsal up. Wholemount images show dorsolateral views with anterior to the left.

**Figure 7:**
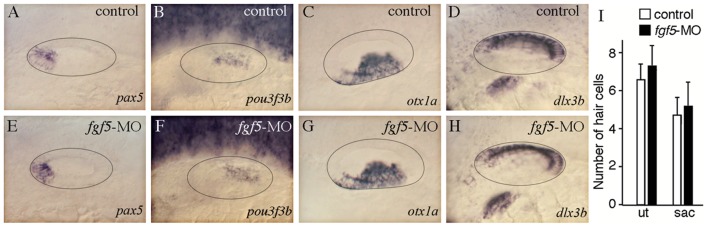
Normal axial patterning in *fgf5* morphants. (A–H) Expression of regional patterning markers in control embryos (A–D) and *fgf5* morphants (E–H). Expression of *pax5* (A, E) and *pou3f3b* (B, F) labels anterior and posterior regions, respectively. Expression of *otx1a* (C, G) and *dlx3b* (D, H) labels ventromedial and dorsolateral regions, respectively. The otic vesicle is outlined. Images show dorsolateral views with anterior to the left. (I) The total number of hair cells in the utricular (ut) and saccular (sac) maculae of control embryos and *fgf5* morphants at 32 hpf. Data were obtained by counting GFP-positive hair cells (mean of total number ± standard deviation) in the sensory epithelia of *brn3c:Gfp* transgenic embryos. Data show means and standard deviations from 20 specimens each. Differences between control and experimental specimens were not statistically significant (p = 0.16 for the utricle, p = 0.67 for the saccule) based on Student's *t* tests.

To address the role of *fgf5* in neuroblast specification we examined *neurog1* expression at various stages in *fgf5* morphants. At 24 hpf no obvious difference was observed between *fgf5* morphants and control embryos (not shown). By 30 hpf, however, *neurog1* expression was dramatically expanded in *fgf5* morphants (Figure 6A, 6B, 6E, 6F), including a pronounced mediolateral expansion of *neurog1* in the floor of the otic vesicle (Figure 6B, 6F). Normally, neuroblast specification declines dramatically after 30 hpf [1], [20] (Figure 6C). However, *fgf5* morphants continued to show abundant *neurog1*-positive cells at 36 hpf, indicating a prolonged phase of robust specification and delamination (Figure 6G). Neuroblast specification/delamination finally ceased by 48 hpf in *fgf5* morphants (not shown). Knockdown of *fgf5* appeared to affect SAG development in a highly specific manner, as other regional markers in the otic vesicle were expressed normally and development of sensory hair cells was also normal at 32 hpf (Figure 7). Additionally, the *fgf5* morphant phenotype was rescued by strong activation of *hs:fgf8* (39°C) at 24 hpf such that neuroblast specification returned to normal (Figure 6D, 6H). Such rescue supports the idea that neuroblast specification relies on a proper balance of Fgf signaling, with the morpholino and transgene counter-balancing each other. Overall, these data (summarized in Table 1) support the hypothesis that mature SAG cells become a source of elevated Fgf, which eventually exceeds a signaling threshold that serves to terminate neuroblast specification in a timely manner.

To test this model in another way, mature neurons marked by *isl2b:gfp* transgene expression [22] were killed by serial laser-ablation at 22 hpf and 25 hpf (Figure 6I, 6J) and *neurog1* expression was examined at 30 hpf. Expression of *neurog1* was expanded on the ablated side relative to the unablated (contralateral) side (Figure 6K, 6L, Table 1). Together, these data support the notion that as mature neurons expressing *fgf5* accumulate within the SAG, overall levels of Fgf signaling increase and as a result neuroblast specification is terminated. This also explains the increased susceptibility to misexpression of Fgf8 after 24 hpf, as described above.

### Fgf regulates the balance between transit-amplification and differentiation

We next examined the effects of Fgf on post-delamination stages of SAG development. In these experiments heat shock transgenes were activated at high levels (38–39°C) at 24 hpf and the effects on *neurod* ^+^ (transit-amplifying) and Isl1^+^ (mature) populations were examined at 36 hpf and 48 hpf. Summing *neurod* ^+^ cells in serial sections of control embryos indicated that there are approximately 200–250 transit-amplifying cells in the SAG at these stages (Figure 1B). Because this approach proved laborious and was prone to occasional loss of tissue sections, changes in the *neurod* domain were assessed by measuring mean cross-sectional areas in the three AP regions of the SAG in transgenic and control embryos.

**Figure 8:**
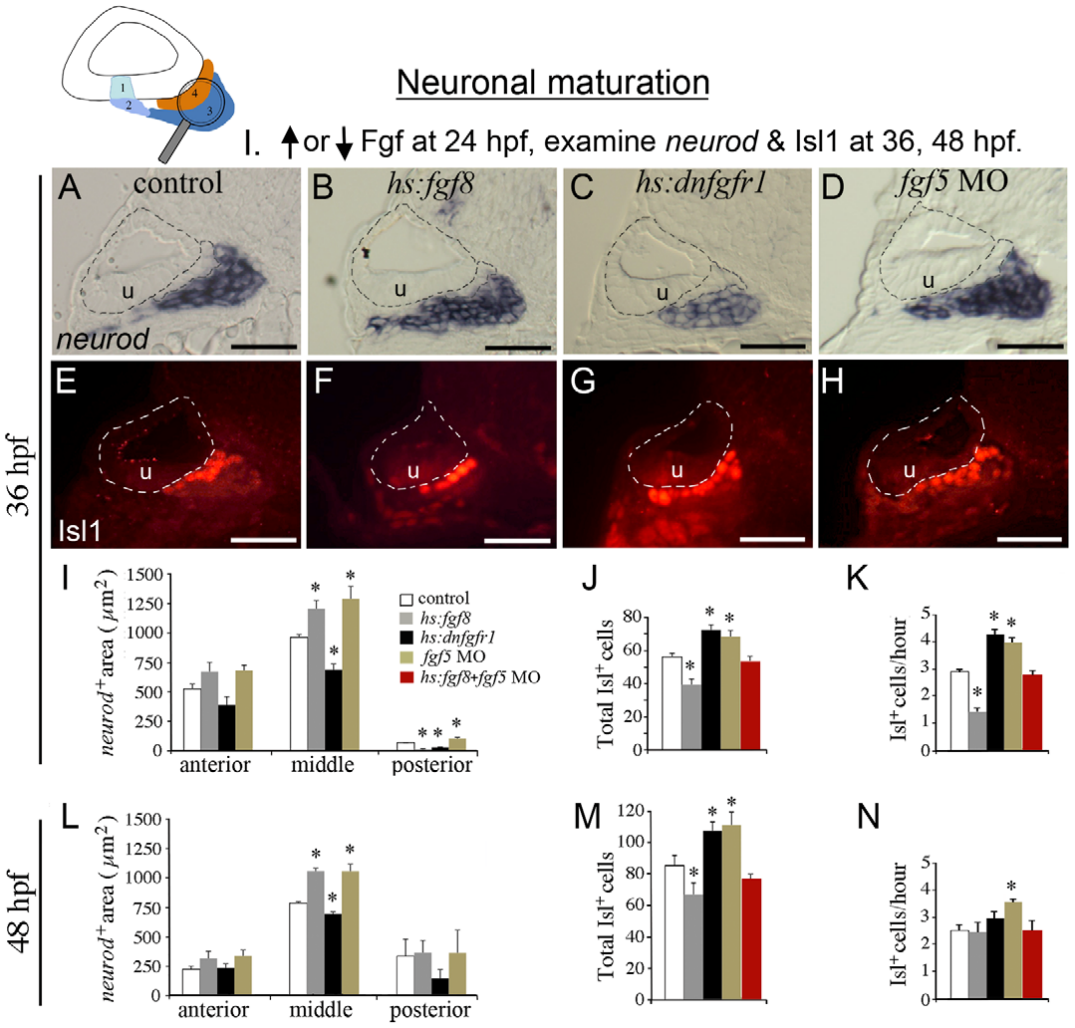
Fgf regulates the balance between transit-amplification and differentiation. The icon at the top of the figure indicates that neuronal maturation (*neurod* ^+^ transit-amplifying cells and Isl1^+^ mature neurons) is the focus of analysis. Manipulations in these experiments (Neuronal maturation group I) are briefly summarized at the top. Embryos were heat shocked for 30 minutes at 39°C (wild-type controls, *hs:fgf8/+* embryos, and *fgf5-*morphants) or 38°C (*hs:dnfgfr1/+* embryos) beginning at 24 hpf. (A–H) Transverse sections (lateral to the left, dorsal up) showing *neurod* expression (A–D) or Isl1 staining (E–H) at 36 hpf in control embryo (A, E), *hs:fgf8/+* embryos (B, F), *hs:dnfgfr1/+* embryos (C, G) and *fgf5* morphants (D, H). All sections shown pass through the middle region of the SAG at the level of the utricular macula (u). The otic vesicle is outlined. Scale bar, 25 µm. (I–N) Quantitation of transit-amplifying and mature neuronal populations at 36 hpf (I–K) and at 48 hpf (L–N). Panel I shows a color key to facilitate comparison between treatments: White bars, control; gray bars, *hs:fgf8*; black bars, *hs:dnfgfr1*; brown bars, *fgf5* morphants; red bars, activation of *hs:fgf8* in *fgf5* morphants. Analysis of transverse sections was used to measure the mean area of *neurod^+^* precursor cells (I, L) in the anterior, middle and posterior regions of SAG. The total number of Isl1+ neurons (J, M) and the mean hourly rate of neuron production from 24 hpf to 36 hpf (K) and from 36 to 48 hpf (N) was measured by counting neurons in stained wholemount specimens. Error bars in I, J, L, M indicate standard deviations (n = 3 or greater for sectional areas; n = 15 for Isl1^+^ cell counts). *p<0.05 in comparison to control, analyzed with Student's *t* test. Error bars in K, N indicate standard errors.

Strong activation of *hs:fgf8* at 24 hpf (39°C for 30 minutes) increased the *neurod* ^+^ precursor domain by 31% in the largest, middle region of the SAG at 36 hpf (Figure 8A, 8B, 8I). A similar trend was observed in the anterior region, although the difference was not statistically significant (Figure 8I). Under these conditions, the smallest, posterior part of the SAG was truncated in *hs:fgf8* embryos and therefore was nearly devoid of *neurod* ^+^ cells in most specimens (Figure 8I). This is possibly because the posterior SAG forms later and elevated Fgf prematurely terminates specification of neuroblasts that might otherwise contribute to this region. Despite, the increased population of transit-amplifying cells in the middle region, the total number of Isl1^+^ neurons in the SAG was reduced in *hs:fgf8* embryos by 30% at 36 hpf (Figure 8E, 8F, 8J) and the hourly rate of neuron production between 24 hpf and 36 hpf was reduced by half (Figure 8K). For loss of function studies, *hs:dnfgfr1* was activated at 24 hpf (38°C for 30 minutes) to impose a strong block to Fgf signaling. This resulted in a decrease of 26% in the *neurod* ^+^ domain in the middle region at 36 hpf, and a decrease of 50% in the posterior region (Figure 8C, 8I). Again, the anterior region showed a similar but non-significant trend. Under the same conditions, there was a 30% increase in the total number of mature Isl1^+^ SAG neurons (Figure 8G, 8J). The relative effects of *hs:fgf8* and *hs:dnfgfr1* on the transit-amplifying population persisted through at least 48 hpf (Figure 8L). Differences in the total number of mature neurons also persisted at 48 hpf (Figure 8M). However, most of the differences seen at 48 hpf appeared to reflect changes occurring before 36 hpf because the rate of production of new Isl1^+^ neurons after 36 hpf was nearly normal in *hs:fgf8* and *hs:dnfgfr1* embryos (compare Figure 8K, 8N). This presumably reflects the transient nature of transgene activity and gradual reestablishment of normal SAG regulation. Note that under the conditions used here, we detected little or no cell death in the transit-amplifying or mature regions of the SAG as shown by staining with Acridine Orange or anti-Caspase 3 antibody (not shown). Likewise, we detected no changes in the number of mitotic cells in the SAG, nor in the proportion of cells incorporating BrdU (data not shown), indicating that Fgf does not directly affect cell cycle dynamics. Instead, the data (summarized in Table 2) suggest that Fgf slows the rate at which transit amplifying cells differentiate into mature SAG neurons, whereas blocking Fgf accelerates differentiation.

**Table 2:** Effects of altering Fgf on SAG maturation.

Condition and stage	Size *neurod* domain	Number Isl1^+^ cells	Figure
*hs:fgf8*-high (39°C), 24 hpf	increased 36, 48 hpf	reduced 36, 48 hpf	8B, 8F, 8I–8N
*hs:dnfgfr1*-high (38°C), 24 hpf	reduced 36, 48 hpf	increased 36, 48 hpf	8C, 8G, 8I–8N
*fgf5*-MO, 1-cell	increased 36, 48 hpf	increased 36, 48 hpf	8D, 8H, 8I–8N
*fgf5*-MO+*hs:fgf8* (39°C), 24 hpf	not determined	normal 36, 48 hpf	8J, 8K, 8M, 8N
Ablate mature SAG 30, 32 hpf	reduced 44 hpf normal 56 hpf	normal 34–44 hpf* increased 56–80 hpf*	9A
*hs:fgf8*-high (39°C), 34 hpf	normal 56 hpf	reduced 34–56 hpf*	9B
*hs:dnfgfr1*-high (38°C), 34 hpf	normal 56 hpf	increased 34–56 hpf*	9B
Ablate SAG+*hs:fgf8* (39°C), 34 hpf	normal 56 hpf	normal 34–56 hpf*	9B
Ablate SAG+*hs:dnfgfr1* (38°C), 34 hpf	normal 56 hpf	increased 34–44 hpf*	9B

* Rate of mature neuron production during the indicated interval.

We next assessed the role of Fgf5 in restraining maturation of precursor cells. In *fgf5* morphants, the size of *neurod* ^+^ domain was increased in both the middle and posterior regions of SAG in the embryos at 36 and 48 hpf (Figure 8D, 8I, 8L). Note that the increase in the transit-amplifying region seen in *fgf5* morphants was different from what was observed following activation of *hs:dnfgfr1*. This is presumably because the prolonged phase of robust specification seen in *fgf5* morphants (Figure 6G, 6H) continues to replenish the transit-amplifying population. Additionally, *fgf5* morphants also produced more Isl1^+^ neurons than normal (Figure 8H, 8J, 8K, 8M, 8N). However, despite the enlarged pool of precursors *fgf5* morphants did not produce more mature neurons than did *hs:dnfgfr1* embryos (Figure 8J, 8M). This is possibly because redundant factors (possibly macular Fgfs) continue to restrain the enlarged pool of progenitors in *fgf5* morphants. Finally, we observed that strong activation of *hs:fgf8* (39°C) at 24 hpf in *fgf5*-morphants restored neuron production to normal (Figure 8J, 8K, 8M, 8N). Thus, as during neuroblast specification, the rate of neuronal maturation is also regulated by a proper balance of Fgf signaling. Moreover, these data (summarized in Table 2) support a role for Fgf5 as a feedback inhibitor released by mature SAG neurons to restrict the rate of neuronal differentiation.

### Neuronal maturation following ablation of mature SAG neurons

**Figure 9:**
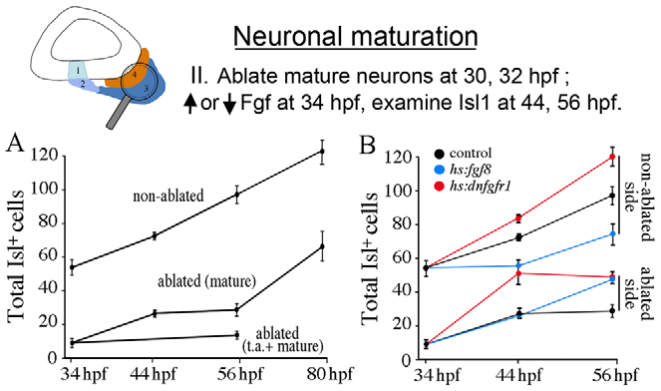
Regeneration following SAG ablation. The icon at the top of the figure indicates that neuronal maturation is the focus of analysis. Manipulations in these experiments (Neuronal maturation group II) are briefly summarized at the top. (A) Accumulation of Isl1^+^ SAG neurons in *isl2b:Gfp/+* embryos after serial ablation of Gfp-positive neurons (mature) or ablation of Gfp-positive neurons and transit-amplifying cells (t.a. + mature) at 30 hpf and 32 hpf. Neuronal accumulation on the contralateral (non-ablated) side served as a control. (B) Effects of modulating Fgf after serial ablations at 30 hpf and 32 hpf on the total number of Isl1^+^ neurons. Embryos were heat shocked for 30 minutes at 39°C (+/+ and *hs:fgf8/+* embryos) or 38°C (*hs:dnfgfr1/+* embryos) beginning at 34 hpf. Data show means and standard deviations of 2–5 specimens per time point.

To further explore regulation of SAG maturation, we assessed whether laser-ablation of mature SAG neurons affects the rate of new neuron production. This analysis was conducted after 30 hpf to minimize the impact of neuroblast specification on overall cell number. Using the *isl2b:Gfp* line, mature SAG neurons on one side of the head were targeted for serial ablation at 30 hpf and 32 hpf, with the contralateral side serving as a non-ablated control. We observed that a single round of ablation was inefficient, allowing a substantial fraction of neurons to survive. However, serial ablation successfully eliminated over 90% of mature neurons, as confirmed by anti-Isl1 staining just after the second ablation (not shown). Analysis of the transit-amplifying population revealed that the number of *neurod^+^* cells declined by 10–20% on the ablated side during the first 12 hours following neuronal ablation, probably reflecting collateral damage, but the number returned to normal by 56 hpf (24 hours post-ablation) (data not shown). Despite the initial decrease in transit-amplifying cells, new Isl1^+^ neurons accumulated at a rate comparable to the non-ablated contralateral side for the first 12 hours after ablation, (Figure 9A). The rate of neuron production briefly declined during the next 12-hour period, but then increased to a rate 60% greater than normal through at least 80 hpf (Figure 9A, Table 2). Co-ablation of both mature and transit-amplifying cells (the latter were targeted based on position and morphology) nearly eliminated production of new Isl1^+^ cells through at least 56 hpf (Figure 9A), confirming the vital importance of transit-amplifying cells for producing new mature SAG neurons. Together these data suggest that loss of feedback inhibition from mature neurons leads to accelerated differentiation of cells from a pool of self-renewing progenitors.

We next examined whether altering Fgf signaling influences neuron production after 30 hpf, with and without laser-ablation of mature neurons. Ablations were conducted in transgenic embryos carrying both *isl2b:Gfp* and either *hs:fgf8* or *hs:dnfgfr1*. Again, *isl2b:Gfp* ^+^ cells were serially ablated on one side at 30 hpf and 32 hpf, and embryos were then heat shocked at 38°C or 39°C (strong activation) at 34 hpf. The contralateral side served as a non-ablated control. On the non-ablated side, the effects of activating *hs:fgf8* or *hs:dnfgfr1* at 34 hpf were similar the effects of activating these transgenes at 24 hpf: Specifically, strongly elevating Fgf impaired production of new neurons whereas blocking Fgf accelerated production of new neurons (Figure 9B, Table 2). On the ablated side, activation of *hs:dnfgfr1* (38°C) accelerated production of new neurons to more than twice the normal rate through 44 hpf, after which the rate flattened out as in non-transgenic ablations (Figure 9B). Moreover, the rate of neuron production in ablated *hs:dnfgfr1* embryos was 50% greater than in non-ablated *hs:dnfgfr1* embryos. Surprisingly, strong activation of *hs:fgf8* (39°C) resulted in a rate of neuronal accumulation on the ablated side that was nearly normal (comparable to the non-ablated control). Thus, misexpressing Fgf8 counterbalances the effects of eliminating mature neurons (and hence Fgf5) such that there is no net change in the rate of neuron production. This is similar to the ability of *hs:fgf8* to counterbalance the effects of *fgf5*-MO on neuroblast specification (Figure 6H) and maturation of SAG neurons (Figure 8J, 8K, 8M, 8N). Analysis of the *neurod* ^+^ domains showed that transgene activity had no significant effect on the size of the transit-amplifying pool at these stages (Table 2). Thus, blocking Fgf accelerates production of new neurons and enhances the effects of neuronal ablation whereas misexpressing Fgf8 offsets the effects of neuronal ablation (summarized in Table 2). These data further support the hypothesis that Fgf5 from mature neurons acts as a feedback inhibitor to slow the rate of maturation of new SAG neurons.

## Discussion

**Figure 10:**
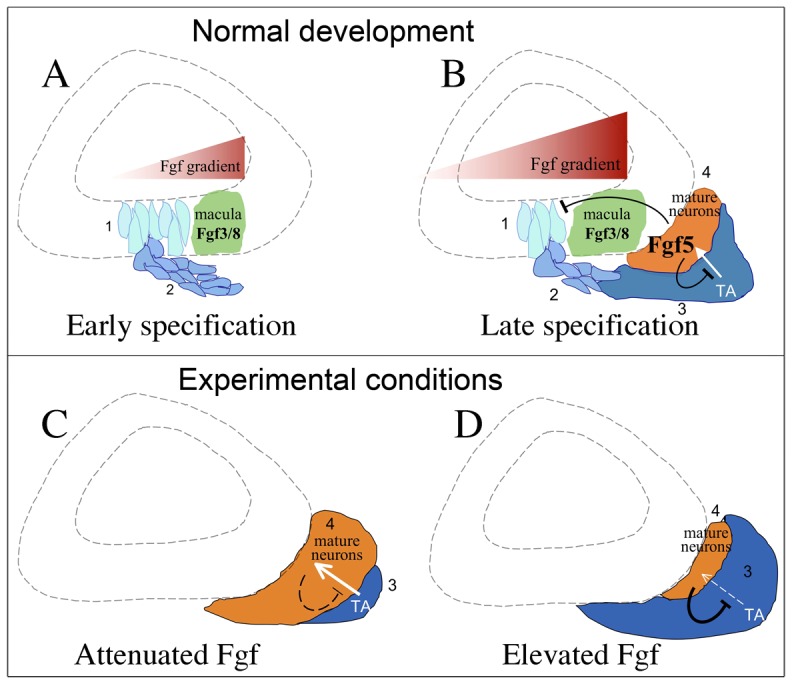
Model for regulation of SAG development by Fgf. (A) Neuroblast specification at early stages. A moderate level of Fgf3 and Fgf8 in a gradient generated by the utricular macula specifies neuroblasts in the floor of the otic vesicle (step 1), and nascent neuroblasts quickly delaminate from the otic vesicle (step 2). (B) As development proceeds, neuroblasts establish a pool of transit-amplifying (TA) progenitors (step 3), which eventually differentiate into mature neurons and express Fgf5 (step 4). Rising levels of neuronal Fgf5, combined with Fgf3 and Fgf8 from the growing utricular macula, exceeds an upper threshold that serves to terminate specification of new neuroblasts within the otic vesicle. Neuronal Fgf5 also slows differentiation of progenitors into mature neurons. (C, D) At stages immediately following establishment of the transit-amplifying pool, experimental attenuation of Fgf signaling promotes maturation of neurons at the expense of progenitors (C) whereas elevating Fgf inhibits maturation, expanding the size of the transit-amplifying pool (D).

The data presented here support a model in which changing levels of Fgf differentially regulates distinct stages of SAG development (Figure 10). Initially a moderate level of Fgf in a spatial gradient specifies the neurogenic domain within the otic vesicle. Subsequently, Fgf levels gradually rise as differentiating SAG neurons accumulate and express Fgf5, eventually terminating neurogenesis in the otic vesicle. It is likely that the expanding macular source of Fgf also contributes to termination of neurogenesis. Terminal differentiation of SAG neurons initially occurs rapidly following delamination from the otic vesicle. However, the developmental increase in Fgf delays neuronal differentiation, maintaining the transit-amplifying phase. This allows the developing SAG to achieve a steady state in which the rate of progenitor growth just matches the rate of neuronal differentiation. This property of the SAG is presumably necessary to provide sufficient neurons to innervate growing sensory epithelia, which continue to expand throughout larval and early adult stages in zebrafish [30]. Knockdown of *fgf5* prolongs the phase of neuroblast specification and also accelerates the rate of neuronal differentiation. Neuroblast specification eventually ceases in *fgf5* morphants, possibly in response to elevated Fgf from the growing utricular macula. We cannot assess the long-term effects of *fgf5* knockdown because morpholino efficacy dissipates after 3–5 days. However, once specification/delamination ceases, accelerated neural differentiation in the absence of *fgf5* function would be expected to deplete the transit-amplifying pool, leading to a neural deficiency in the long-run.

Cell cycle dynamics are likely to be rather complex in the transit-amplifying population. With a steady state of 180–200 progenitors producing 50–60 neurons per day, the average cell cycle length could be unusually long assuming that most progenitors continue to cycle. However, patterns of BrdU incorporation suggest cell cycle dynamics are not uniform amongst progenitors. A 6-hour incorporation period labels roughly 40% of *neurod* ^+^ progenitors, with labeling being especially prominent in distal regions of the SAG (Figure 1E, 1F, and data not shown). It is therefore possible that a substantial fraction of progenitors enter a quiescent state. We detected no overt effect of Fgf signaling on the pattern of BrdU incorporation or the incidence of mitotic cells (not shown), although we cannot exclude the possibility that our heat shock lines act too transiently to detectably alter cell cycle dynamics. Nevertheless, modulating Fgf clearly had a rapid effect on the rate of production of mature neurons (Figure 8 and Figure 9), thereby indirectly affecting the progenitor pool. Interestingly, activating *hs:fgf8* or *hs:dnfgfr1* at 24 hpf caused lasting changes in the size of the progenitor pool (Figure 8), whereas no such change was seen when the transgenes were activated at 34 hpf (data not shown). This difference likely reflects the greater relative impact of changing the rate of neuronal differentiation at early stages when the progenitor pool is still small. In contrast, with nearly 200 progenitors at 32 hpf, it is not surprising that altering the rate of neuronal differentiation by 1–2 cells per hour for several hours had little impact on the progenitor pool.

Although the model in Figure 10 depicts the influence of macular Fgfs on SAG specification in the otic vesicle, it must be emphasized that a lateral gradient of Fgf from the hindbrain acts much earlier to coordinate formation of sensory and neural progenitors in adjacent domains during placodal stages. The prosensory gene *atoh1b* is induced at 10.5 hpf in the nascent otic placode in medial cells closest to the Fgf-source [13], and elevating Fgf expands this domain laterally (our unpublished observations). Expression of *neurog1* begins in more lateral tissue by 16 hpf (14 somites) and is initially influenced by the same hindbrain source of Fgf [15], [16]. We have confirmed here that neuroblast specification requires Fgf whereas excess Fgf inhibits *neurog1* expression, consistent with the notion that the neurogenic domain is established by intermediate levels of Fgf in a diffusion gradient. After formation of the otic vesicle, the utricular macula provides the strongest source of Fgf in the otic vesicle and the neurogenic domain forms an arc wrapping around the lateral and posterior edges of this source [20]. Later the saccular macula begins to expand and expresses more Fgf [13], [31], with a corresponding posterior extension of the neurogenic domain to form a narrow band just lateral to the saccule [20]. Soon thereafter Fgf levels exceed the upper threshold and terminate neuroblast specification.

In zebrafish, it is currently unclear whether the spatial distribution of neuroblasts within the otic vesicle is directly tied to later distribution of mature neurons in the SAG. However, fate mapping studies in chick and mouse reveal that the spatial-temporal progression of neuroblast formation in the otic vesicle presages the spatial-temporal accumulation of vestibular and auditory neurons outside the ear [32], [33]. It is likely that a similar progression occurs in zebrafish, though fate mapping studies have yet to confirm this relationship. Mechanism of SAG subtype specification has not been well characterized in any species, though several studies in zebrafish suggests that Shh is involved. Disruption of Hedgehog signaling ablates posterior fates in the otic vesicle, including the saccule and posterior (presumptive auditory) neurons [23], [34]. Fgf signaling acts in opposition to Shh by promoting anterior fates in the otic vesicle [15], [16], [34], though altering Fgf does not appear to cause wholesale redistribution of SAG neurons. However, we found that activating *hs:fgf8* at 24 hpf caused premature termination of neuroblast specification and also blocked later production of posterior/auditory neurons, supporting a link between spatial/temporal cues and SAG subtype-specification.

### Are the distinct roles of Fgf conserved in amniotes?

Numerous studies support a role for Fgf in SAG neuroblast specification in the chick and mouse. In chick, misexpression of Fgf8 or Fgf10 during placodal stages causes expansion of the neurogenic domain in the otic vesicle, whereas blocking Fgf signaling dramatically reduces the neurogenic domain [4], [35]. In mouse, knockout of *Fgf3* or receptor isoform *Fgfr-2 (IIIb)* causes severe deficiencies of delaminating neuroblasts and neurons [11], [36]. Explant cultures of chick or mouse otocysts treated with exogenous Fgf2 produce 5- to 10-fold more delaminated neuroblasts compared to controls, whereas blocking Fgf2 with a neutralizing antibody severely reduces the number of neuroblasts [10], [37]. Thus the requirement for Fgf in neuroblast specification appears broadly conserved. However, the spatial gradient of Fgf that we propose coordinates sensory and neural development in zebrafish is unlikely to operate in mammals. Unlike the situation in zebrafish, in mouse the neurogenic and sensory domains overlap spatially but are specified at slightly different times. Neuroblast specification occurs first, but as the phase of neuroblast specification/delamination begins to wane sensory epithelia begin to form in the same region. The transition from neural to sensory development partly reflects mutual repression between Neurog1 and Atoh1, the principal initiators the proneural and prosensory pathways, respectively [38]. Whether Fgf also influences this transition is not known.

Despite the above studies showing a requirement for Fgf, it is not clear whether high levels of Fgf are inhibitory in birds and mammals as we have shown here, nor whether Fgf delays maturation of cells in the transit-amplifying pool. In explants of chick or mouse otocysts, exposure to Fgf accelerates the appearance of mature neurons compared to cultures lacking exogenous Fgf [10], [37]. At first glance, these results appear to contradict our findings that Fgf delays differentiation. However, Fgf levels used in the above explant studies were based on dose-response curves and were selected to optimize growth of the explant. Hence potential inhibitory effects of higher doses of Fgf were not evaluated. Furthermore, neuroblasts in culture disperse after delamination rather than accumulating against the otocyst wall where they might facilitate feedback inhibition. This possibly explains why otic explants continue to produce neuroblasts for many days, far longer than during normal embryonic development. In rodent embryos, differentiating auditory neurons express Fgf1, Fgf2, Fgf5 and Fgf10 [36], [39]–[42], which could help mediate feedback inhibition. Unfortunately, relevant functional studies are lacking. In adult rodents, neuronal Fgf is thought to play a role in maintenance of the spiral ganglion. Augmenting Fgf mitigates neural degeneration following nerve injury or noise-induced trauma [43], [44]. Additionally, conditional knockout of Fgf receptor genes *Fgfr1* and *Fgfr2* in glial cells in the spiral ganglion leads to progressive loss of auditory neurons beginning around 2 months of age, suggesting a role in promoting trophic support from glia [45]. In cultures of spiral ganglion from adult mouse, exogenous Fgf2 can promote neuronal survival and neurite outgrowth [46]. Unexpectedly, such cultures were also found to contain quiescent progenitors that could be induced to reenter the cell cycle by incubation with EGF and Fgf2, with some cells differentiating into neurons after removal of EGF and Fgf2 [46]. These latter data are consistent with the possibility that Fgf maintains progenitors and inhibits neural differentiation, though it remains to be seen whether such a mechanism operates in vivo.

The developing SAG can be compared to the developing olfactory epithelium (OE). Fgf8 expression around the rim of the olfactory pit stimulates proliferation of OE progenitors, which differentiate into mature neurons deeper inside the pit away from the Fgf8 source [47]. Conditional knockout of Fgf8 results in severe deficiency of neurons due to failure of progenitors to expand. Development of the OE neurons is also regulated by feedback inhibition from mature neurons, though the mechanism differs from the SAG. Specifically, mature OE neurons secrete the TGFβ factor GDF11, which inhibits further proliferation of progenitors by antagonizing Fgf8 [48]. In the eye, too, GDF11 acts as a feedback inhibitor of retinal ganglion cells, though in this case GDF11 blocks further differentiation of progenitors rather than restricting proliferation [49].

In numerous other settings, Fgf regulates the balance between growth and differentiation of neural progenitors. In cultures of human or rat cortical progenitors, high levels of Fgf stimulate proliferation and block neuronal differentiation [50], [51]. In the developing midbrain-hindbrain region in mouse, conditional knockdown of Fgf receptors results in an increase in differentiated neurons and a concomitant loss of progenitor cells in the ventricular zone [52]. During earlier stages of mouse development, Fgf induces embryonic stem (ES) cells to form epiblast, which begin to express Fgf5. Subsequently, Fgf maintains the epiblast as a stable intermediate by preventing reversion back to the ES ground state and blocking further differentiation into neural ectoderm [53]. Thus, maintenance of stable progenitor pools by Fgf appears to be a broadly conserved mechanism utilized in many aspects of neural development. A relatively novel aspect of SAG development is that Fgf coordinates the entire process, initially specifying neuroblasts and, at a higher level, also mediates feedback from mature neurons to inhibit further differentiation. How changing levels of Fgf achieve this balance remains an important unresolved question.

## Materials and Methods

### Fish strains, misexpression, and inhibitor treatment

Wild-type zebrafish strains were derived from the AB line (Eugene OR). The following transgenic lines were used in this study: *Tg(hsp70:fgf8)^x17^* [24], *Tg(hsp70I:dnfgfr1-EGFP)^pd1^* [25] and *Tg(−17.6isl2b:GFP)^zc7^* [22]. Embryos were maintained at 28°C, unless otherwise stated, and staged according to standard protocol [54]. Heat shock-inducible transgenes were activated by incubating embryos for 30 minutes at elevated temperatures as indicated in the Results. In some experiments, Fgf signaling was blocked by treating wild-type embryos in their chorions with SU5402 (Tocris Bioscience) diluted from a 20 mM stock in DMSO to a final concentration of 100 µM SU5402. PTU (1-phenyl 2-thiourea, 0.3 mg/ml, Sigma) was added to fish water to prevent melanin formation.

### Morpholino injection and RT–PCR

To block *fgf5* translation, we used *fgf5tb*-MO” 5′-CATTCTTTCCAGAGAGCGCTAGGCC-3′. To block splicing of *fgf5* transcript, we used *fgf5i1e2*-MO: 5′ -GCTCCAGCACACCTAGATAGAGAAA- 3′. Approximately 5 ng morpholino was injected per embryo at one-cell stage. Both morpholinos gave identical phenotypes. The efficacy of the splice blocker was assessed at 24 hpf by RT-PCR with primers P1 (forward), 5′-TCGATGGAAGAGTCAACGGGAGC-3′ and P2 (reverse) 5′-GCCTTCCCCTCTTGTTCATGGC-3′ (see Figure 4D, 4E). Expression of *ornithine decarboxylase* (*odc*) was measured as a constitutive control. Uninjected embryos from the same genetic background were used to measure control transcript levels.

### *In situ* hybridization

Whole-mount *in situ* hybridization was carried out with methods described previously [55], [56]. A shorter riboprobe was synthesized for *neurog1* using T7 RNA polymerase to avoid binding to shared vector sequences encoded by the *Tg(−17.6isl2b:GFP)^zc7^* transgene. To improve signal and reduce background staining during *in situ* hybridization for *fgf5*, pre-hybridization and hybridization were performed at 70°C for 12 hours and 24 hours, respectively.

### Immunostaining

Antibody staining was performed as described previously [57]. Primary antibodies were as follows: anti-Islet1/2 (Developmental Studies Hybridoma Bank 39.4D5, 1∶100 for whole-mount, 1∶250 for cryosections) and anti-BrdU (Beckton-Dickinson, 1∶300). Secondary antibodies were as follows: HRP-conjugated goat anti-mouse IgG (Vector Labs PI-2000, 1∶200) and Alexa 546 goat anti-mouse IgG (Invitrogen A-11003, 1∶250).

### Cryosectioning and BrdU labeling

Fixed embryos were washed three times for 5 min each in 1× PBS and then soaked in 20% sucrose solution made in PBS followed by 30% sucrose until sinking to the bottom of a microcentrifuge tube. Embryos were embedded in tissue freezing medium (Triangle Biomedical Sciences, TFM-C) and transverse sections were cut at 10 µm thickness using a cryostat and immunostained. Finally, slides were washed twice in 1× PBS and mounted in ProLong Gold (Invitrogen) with a coverslip. For double labeling, whole-mount *in situ* hybridization was performed first followed by immunostaining on cryosections. For BrdU labeling, dechorionated embryos were incubated in fish water containing 10 mM BrdU in 1% DMSO for the indicated duration. Embryos were rinsed twice for 5 minutes each in fish water prior to fixation. For older stages (96 hpf) 2 nl of 10 mM BrdU/1% DMSO solution with 3% filtered green food coloring was injected into the brain ventricle of larvae anesthetized in Tricaine (Sigma). Embryos were first processed by whole-mount *in situ* hybridization for *neurod* and then cryosectioned. Slides were washed thrice for 5 minutes each in PBT (with 0.1% Triton) and incubated in 2N HCl for 45 minutes at 37°C. Slides were rinsed in PBT again, incubated in blocking solution (with 1% Triton for 36 hpf and 3% Triton for 102 hpf) for 2 hours and stained for BrdU.

### Laser ablation

Maturing SAG neurons were ablated using a MicroPoint laser, under 40× objective, in *isl2b:GFP* transgenic line that labels this population of cells. Anesthetized embryos were mounted in a dorsolateral orientation beneath a #1 coverslip on a bridge slide made by stacking two #1 coverslips on either side of the embryo.
